# Structural and Practical Departments

**Published:** 1906-07-28

**Authors:** 


### STRUCTURAL AND PRACTICAL DEPARTMENTS.

### BOYLE'S AIR-PUMP VENTILATORS.

The name of Boyle is a household word in connection with what is termed "natural" as distinguished from mechanical ventilation. Mr. Robert Boyle and his sons, have consistently maintained that efficient ventilation can be obtained by exhausting the hot air and gases from the upper part of the room, with the aid of some apparatus, similar to theirs, and they claim to have been uniformly successful in competition with every other kind of ventilating apparatus—the Grand Diploma with prize of £50 having been awarded to them at the International Ventilation Competition held in London. The Russian Government are employing Messrs. Boyle's system of ventilation with their air-pump ventilator, in the Government buildings of St. Petersburg, and also in a number of men-of-war now in course of construction. H.M. ship *Hardinge*, a large Indian trooper, has been fitted with Boyle's apparatus, as well as some of the North German Lloyd, the Messageries Maritimes, and the Compagnie Generale Trans-Atlantique. The apparatus itself is a very skilful adaptation of the now well-known principle of the eductive effect of air friction. The little apparatus, known as the scent spray, is worked on this principle, as well as some appliances used in connection with steam. When a current of air passes across the open mouth of a pipe, or duct, or chimney, the air or gas in the pipe, or chimney, are sucked upwards, and this is the action of the Boyle's ventilator, with the addition of shields, which protect the chimney or duct from down draughts. The working of the apparatus is very clearly shown by models which Messrs. Boyle have had made, and which exactly represent the actual air-pump ventilator employed by them. The ventilator is placed at the highest possible point, and its duct is connected with the top of the room, either directly, or by means of a pipe leading down the side of the house. It is claimed that Messrs. Boyle's apparatus has been very successful wherever it has been employed. It is very much less expensive to fit up than any system of mechanical ventilation.

### A NEW WARE FOR SANITARY APPLIANCES.

Messrs. Morrison, Ingram and Co., Limited, of the Midway Pottery, Swadlincote, near Burton-on-Trent, and of Manchester, are introducing a new substance for the manufacture of sanitary appliances which they claim is very much stronger than the fire clay that is now almost universally employed. In appearance "Densitas" ware is very similar to the ware made of fireclay and similar substances. It is glazed and prepared in the usual way, finished in either pale buff or white. A test of the strength of the "Densitas" was made at the Whitworth Engineering Laboratory, Owens College, Manchester, last year, with the following result. The breaking strain of fireclay was found to be 330 lbs. per square inch, while that of "Densitas" was 1,184 lbs. per square inch, the latter being three and a half times the strength of the former. This quality, it will be seen, enables the utensils to be made very much thinner and lighter than with fireclay, or, on the other hand, if they are made of the same thickness, they should stand a good deal more knocking about. Lightness in sanitary utensils, combined with strength, is an important quality for hospital work. The makers also claim that the ware is hard and non-porous, even if unglazed, so that it should have the additional qualification that, in case of the glaze chipping off, the aseptic qualities are not lost. It is used for making all the usual appliances for lavatories, operating theatres, wards, bed utensils, etc.

